# Coagulopathy in patients with COVID-19: a systematic review and meta-analysis

**DOI:** 10.18632/aging.104138

**Published:** 2020-11-24

**Authors:** Xiaolin Zhang, Xue Yang, Hongmei Jiao, Xinmin Liu

**Affiliations:** 1 Department of Geriatrics, Peking University First Hospital, Beijing 100034, China

**Keywords:** coronavirus, COVID-19, disease severity, coagulation, coagulopathy

## Abstract

COVID-19 patients frequently exhibit coagulation abnormalities and thrombotic events. In this meta-analysis, we investigated the association between coagulopathy and the severity of COVID-19 illness. Using PubMed, Embase, Cochrane, WanFang Database, CNKI, and medRxiv, a systematic literature search was conducted for studies published between December 1, 2019 and May 1, 2020. We then analyzed coagulation parameters in COVID-19 patients exhibiting less severe and more severe symptoms. All statistical analyses were performed using Stata14.0 software. A total of 3,952 confirmed COVID-19 patients from 25 studies were included in the meta-analysis. Patients with severe symptoms exhibited higher levels of D-dimer, prothrombin time (PT), and fibrinogen (FIB) than patients with less severe symptoms (SMD 0.83, 95% CI: 0.70-0.97, I^2^ 56.9%; SMD 0.39, 95% CI: 0.14-0.64, I^2^ 79.4%; and SMD 0.35, 95% CI: 0.17-0.53, I^2^ 42.4%, respectively). However, platelet and activated partial thromboplastin times did not differ (SMD -0.26, 95% CI: -0.56-0.05, I^2^ 82.2%; and SMD -0.14, 95% CI: -0.45-0.18, I^2^ 75.7%, respectively). These findings demonstrate that hypercoagulable coagulopathy is associated with the severity of COVID-19 symptoms and that D-dimer, PT, and FIB values are the main parameters that should be considered when evaluating coagulopathy in COVID-19 patients.

## INTRODUCTION

Coronavirus Disease 2019 (COVID-19) is a viral respiratory infection caused by the 2019 novel coronavirus. As of May 22, 2020, a total of 5,061,476 confirmed cases with 331,475 deaths have been reported globally since the COVID-19 outbreak [1]. Thus, the World Health Organization (WHO) declared the disease a global pandemic. After SARS and MERS, COVID-19 is the third most lethal zoonotic coronavirus disease that occurred in the last two decades [2]; however, it has caused many more deaths than SARS or MERS [3]. Due to the extremely high mortality rate of COVID-19, there is an urgent need to identify the clinical features associated with its progression.

Coagulopathy and thrombotic events are serious complications that occur in some COVID-19 patients. A recent study has reported that the COVID-19 associated coagulopathy is characterized by increased levels of prothrombin time (PT), activated partial thromboplastin time (APTT), and D-dimer [4]. In addition, several studies have shown that elevated D-dimer levels are associated with in-hospital mortality [5, 6]. Recent autopsy reports from the United States have demonstrated that in addition to lung injuries, severe COVID-19 patients exhibit a hypercoagulable state evidenced by thrombotic events in the lung, kidney, and possibly in the heart and other organs [7]. However, whether coagulopathy is associated with the severity of COVID-19 illness remains unclear. Therefore, we conducted a systematic review and meta-analysis by incorporating both English and Chinese published studies to investigate the role of coagulation dysfunction in the severity of COVID-19 progression.

## RESULTS

### Selection of COVID-19 studies

**Figure 1:**
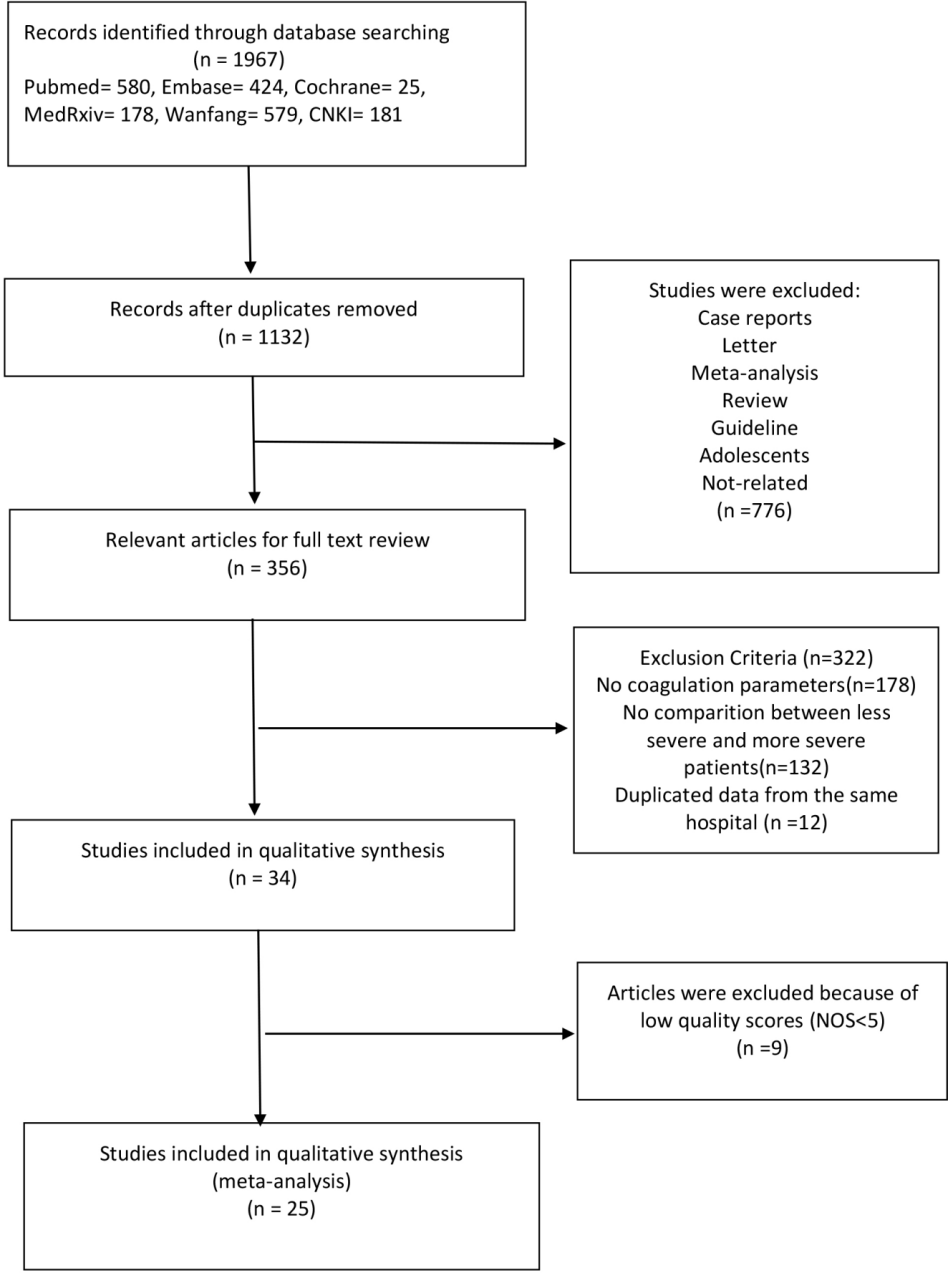
**Schema of literature search.**

The detailed steps of literature search are highlighted in the flow diagram (Figure 1). Using different search strategies and six different databases, 1,967 COVID-19 articles were identified. After eliminating duplicate records, 1,132 articles were obtained. In addition, 776 articles were excluded because they were not original studies or were irrelevant to our meta-analysis. Full texts of the remaining 356 articles were assessed for eligibility, out of which 322 articles were eliminated because they did not meet the exclusion criteria. A total of 34 articles were included in the quality evaluation (Supplementary Table 2), out of which 9 articles were excluded due to a low quality (NOS<5). Finally, 25 articles (1 in Chinese and 24 in English) were selected for the analyses [6, 8–31]. The overall quality of the selected studies was moderate to high, with NOS scores ranging from 5-7.

### Characteristics of the included studies

Data from 3,952 patients in the 25 included studies were used to analyze coagulation parameters in less severe and more severe COVID-19 patients. Notably, 23 studies were conducted in China (11 in Wuhan and 12 in other cities), 1 in Mexico, and 1 in the USA. All studies were retrospective observational studies; sample size varied from 21 to 577. The median age ranged between 46 and 60 years, and the proportion of male patients ranged between 35.3% and 81.0%. Twenty studies followed clinical guidelines (including trial version 4, trial version 5, trial version 7, 6^th^ edition guidelines, 7^th^ edition guidelines, and WHO interim guideline) to evaluate the disease severity. However, two studies considered whether patients experienced ARDS, and one study considered whether patients underwent ICU care to judge the disease severity. The patient characteristics and demographic data for the included studies are shown in Supplementary Table 3.

### Coagulation parameters in less severe and more severe COVID-19 cases

**Figure 2:**
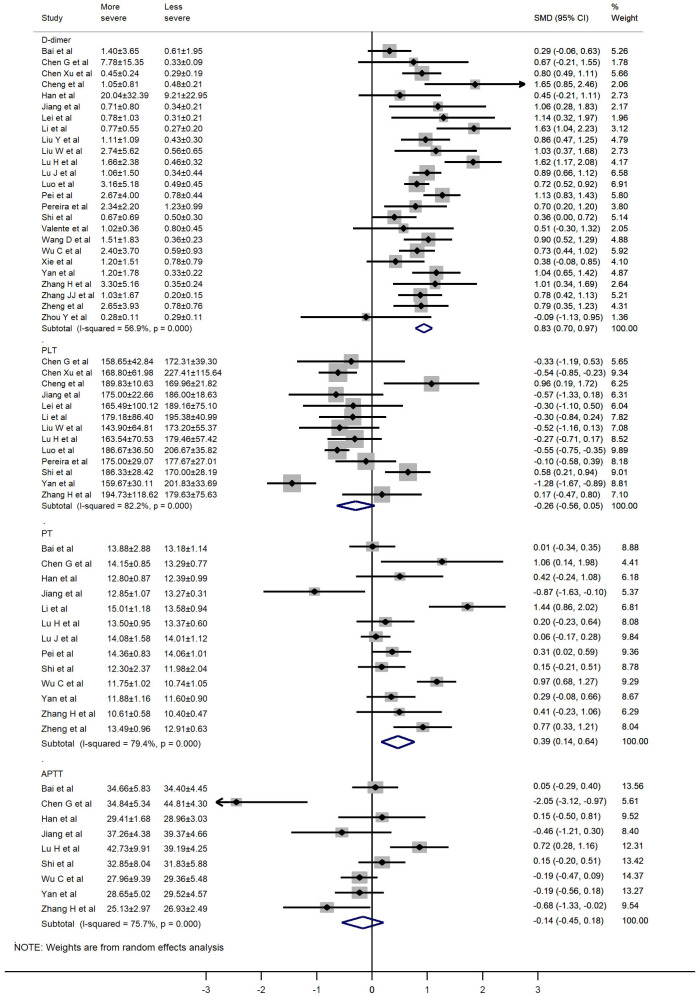
**Forest plot of the association between D-dimer, PLT, PT, and APTT in patients with COVID-19 stratified by disease severity.**

**Figure 3:**
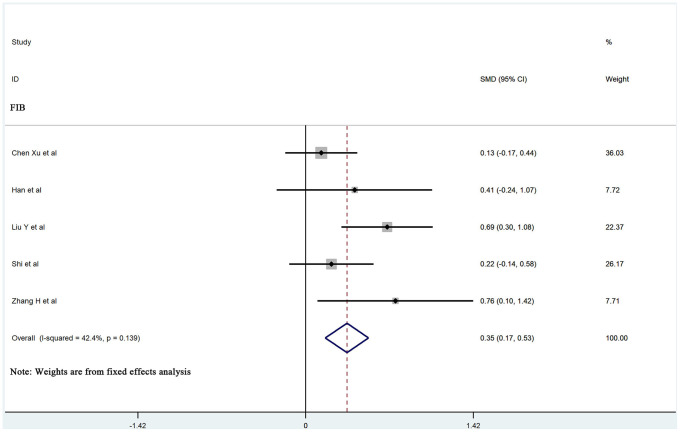
**Forest plot of the association between FIB in patients with COVID-19 stratified by disease severity.**

The values of D-dimer, platelets (PLT), prothrombin time (PT), activated partial thromboplastin time (APTT), and fibrinogen (FIB) were available in 25, 13, 13, 9, and 5 studies, respectively. We used the random-effects model due to significant heterogeneity of D-dimer, PLT, PT, and APTT values. D-dimer and PT values were significantly higher in more severe patients than in less severe patients (SMD 0.83, 95% CI: 0.70-0.97, I^2^: 56.9%; and SMD: 0.39, 95% CI: 0.14-0.64, I^2^: 79.4%, respectively). The fixed-effects model was used due to insignificant heterogeneity of FIB values. Patients with more severe pneumonia exhibited significantly higher FIB values compared to less severe patients (SMD: 0.35, 95% CI: 0.17-0.53, I^2^: 42.4%). However, no significant difference was observed in PLT and APTT values between severe and mild patients (SMD: -0.26, 95% CI: -0.56-0.05, I^2^: 82.2%; and SMD: -0.14, 95% CI: -0.45-0.18, I^2^: 75.7%, respectively). A forest plot of the coagulation parameters is shown in Figures 2 and 3. Details of the meta-analysis are highlighted in Table 1.

**Table 1:** Summary of the meta-analysis results.

		**Test of association**					**Heterogeneity**		
**Groups**	**Studies**	**SMD**	**95%CI**	**p value**	**Model**	**Z**	**Chi-squared**	**p value**	**I^2^(%)**
D-dimer	25	0.83	0.70,0.97	0.000	RE	12.27	55.63	0.000	56.9
PLT	13	-0.26	-0.56,0.05	0.096	RE	1.66	67.27	0.000	82.2
PT	13	0.39	0.14,0.64	0.002	RE	3.04	58.16	0.000	79.4
APTT	9	-0.14	-0.45,0.18	0.384	RE	0.87	32.89	0.000	75.7
FIB	5	0.35	0.17,0.53	0.000	FE	3.75	6.94	0.139	42.4
									
RE, random effects									
FE, fixed effects									
SMD, standardized mean difference									
CI, confidence interval									

### Subgroup analysis

Based on location, severity criteria, and patient age (the median age above and below 50), we conducted a subgroup analysis to determine the sources of heterogeneity. The results of subgroup analysis are presented in Table 2. Notably, the heterogeneity was significant in the subgroup analyses. However, the subgroup analyses did not explain the observed heterogeneity of D-dimer, PLT, PT, and APTT values.

**Table 2:** Results of subgroup meta-analyses.

			**No. of studies**	**SMD (95%CI)**	**P-value**	**Heterogeneity I^2^**	**P value**
D-dimer	Criteria	Guidelines	22	0.85(0.69,1.00)	0.000	61.8	0.000
		ARDS	2	0.77(0.54,1.01)	0.000	0	0.588
		ICU care	1	0.70(0.20,1.20)	0.006	Null	Null
	Location	Wuhan	12	0.76(0.62,0.89)	0.000	42.1	0.061
		Other cities in China	11	1.01(0.72,1.30)	0.000	69	0.000
		Other countries	2	0.65(0.22,1.07)	0.003	0	0.692
	Age	≥50	13	0.72(0.57,0.86)	0.031	48.2	0.026
		<50	9	0.97(0.76,1.17)	0.030	33.8	0.147
PLT	Criteria	Guidelines	12	-0.27(0.60,0.06)	0.104	83.3	0.000
		ICU care	1	-0.10(-0.59,0.39)	0.699	Null	Null
	Location	Wuhan	3	-0.54(-0.73,-0.35)	0.000	0	0.884
		Other cities in China	9	-0.19(-0.65,0.26)	0.407	87	0.000
		Other countries	1	-0.10(-0.58,0.39)	0.699	Null	Null
	Age	≥50	5	-0.26(-0.65,0.14)	0.281	92.1	0.000
		<50	6	-0.34(-0.96,0.28)	0.199	62.3	0.021
PT	Criteria	Guidelines	12	0.36(0.13,0.59)	0.002	69.6	0.000
		ARDS	1	0.91(0.68,1.27)	0.000	Null	Null
	Location	Wuhan	6	0.47(0.13,0.81)	0.070	80.7	0.000
		Other cities in China	7	0.37(-0.03,0.76)	0.068	78.8	0.000
	Age	≥50	6	0.47(0.17,0.76)	0.002	72.9	0.002
		<50	5	0.39(-0.22,1.00)	0.214	87.7	0.000
APTT	Criteria	Guidelines	8	-0.15(0.53,0.23)	0.432	77.8	0.000
		ARDS	1	-0.19(-0.47,0.09)	0.185	Null	Null
	Location	Wuhan	4	-0.30(-0.82,0.22)	0.259	78.8	0.003
		Other cities in China	5	-0.04(-0.48,0.40)	0.862	76.7	0.002
	Age	≥50	6	-0.15(-0.47,0.16)	0.347	69.5	0.097
		<50	1	-0.46(-1.12,0.30)	0.236	Null	Null

### Sensitivity analysis and publication bias

**Figure 4:**
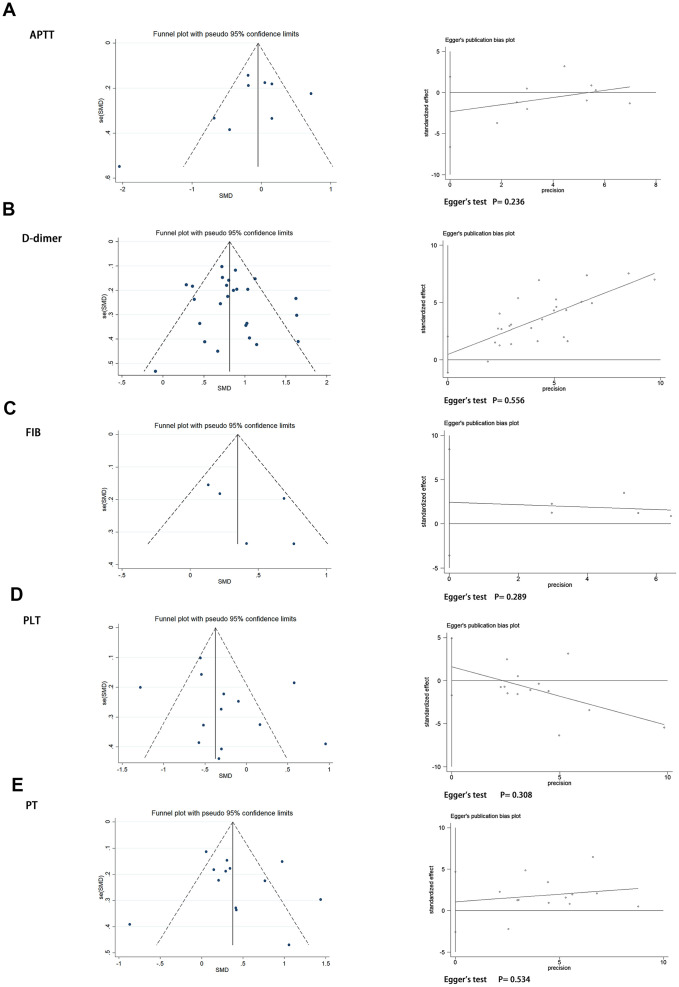
Funnel plot and Egger’s test evaluating the publication bias of (**A**) APTT, (**B**) D-dimer, (**C**) FIB, (**D**) PLT, and (**E**) PT.

Sensitivity analysis showed that the pooled results were not sensitive to any individual study, thus validating the robustness of the study findings. Detailed results of sensitivity analysis are shown in Supplementary Figure 1. In addition, we assessed publication bias using the funnel plots and Egger’s regression test. There was no significant publication bias among the APTT, D-dimer, FIB, PLT, and PT values in our study (Egger test: P = 0.236, P = 0.556, P = 0.289, P = 0.308, P = 0.534, respectively). The funnel diagram and Egger’s test are shown in Figure 4.

## DISCUSSION

Our study demonstrates that coagulopathy is associated with the severity of COVID-19 symptoms, and that the more severe COVID-19 cases are characterized by significantly elevated levels of D-dimer, PT, and FIB. These findings are consistent with conclusions from a previous meta-analysis, which found that PT and D-dimer values were significantly increased in more severe COVID-19 cases [32]. However, we observed no significant decrease in the levels of PLT in severe COVID-19 cases. This observation differs from a previous meta-analysis that included only nine studies, and showed a considerable heterogeneity, possibly leading to unstable results [33].

The increased levels of D-dimer and PT indicate that the fibrinolytic system is activated in severely ill COVID-19 patients. The coagulation cascade is activated in response to viral infections as a host defense mechanism to limit the spread of pathogens. Additionally, increased cytokine release during viral infections stimulates pro-coagulant reactions resulting in coagulation. Furthermore, endothelial cell activation and damage may alter the natural antithrombotic state, given the tropism of the virus for ACE2 receptors [34]. COVID-19 associated coagulopathy (CAC) describes the pro-thrombotic properties in infected patients [35]. Although coagulopathy is reminiscent of disseminated intravascular coagulation (DIC), CAC does not cause clinical bleeding, and is thus distinct from DIC [36]. Clinical observations show that CAC is characterized by elevated fibrinogen, and nearly normal platelet counts [36]; these findings are consistent with our data. This can be explained by extramedullary megakaryocytes within the microvessels that have been detected by autopsy of COVID-19 patients [37]. The megakaryocytes can actively produce platelets within the peripheral circulation [38].

Previous studies have shown that the levels of PT and D-dimer are significantly increased in COVID-19 patients with more severe symptoms, but few studies have analyzed the fibrinogen levels. To our knowledge, this is the first meta-analysis that assessed the correlation between fibrinogen levels and the severity of COVID-19. Several studies have shown that the fibrinogen levels significantly increase during initial and progressive stages of viral infections, but this likely reflects a hypercoagulability instead of a consumptive coagulopathy. Furthermore, a recent study by Ranucci [39] reported comprehensive coagulation analyses on ICU admission with median fibrinogen levels of 7.8 g/L, and increased clot strength through thromboelastometry. SARS-CoV-2 infection often induces a cytokine storm that is dominated by interleukin-6 [40], which stimulates the fibrinogen synthesis. In addition, the variations in fibrinogen levels are dependent on the infection stage. Tang et al [41] reported that the fibrinogen levels decline during the late stages in COVID-19 non-survivors, despite their increased or normal levels on admission; this might be caused by sepsis-induced coagulopathy. Though very few COVID-19 studies have investigated the coagulation cascade in vitro, a related coronavirus, SARS-CoV-1, has been found to upregulate a panel of genes involved in coagulation, including fibrinogen [42].

Since abnormal coagulation parameters including elevated D-dimer and fibrin degradation products are associated with higher COVID-19 mortality rates, monitoring the coagulation parameters is immensely important for managing COVID-19. Zhou et al [5] conducted a study of 191 patients from Wuhan and found that D-dimer levels higher than 1 μg/L were associated with higher in-hospital mortality. WHO recently issued guidelines for managing COVID-19, and suggested that a great attention should be focused on coagulation dysfunction and thrombotic events [43].

Our findings suggest that dynamic monitoring of coagulation parameters in hospitalized COVID-19 patients is necessary for predicting COVID-19 progression with unfavorable outcome and early thrombotic events. Moreover, futures studies should determine whether early anticoagulation treatments, such as low molecular weight heparin (LMWH), warfarin, or new oral anticoagulant (NOAC) are beneficial in COVID-19 patients with significantly elevated coagulation parameters.

Regarding the limitations of our meta-analysis, there was a considerable heterogeneity among the included studies with respect to the definition of COVID-19 severity; both subgroup and sensitivity analyses could not identify the heterogeneity source. Some of the included studies did not use mean and standard deviation; rather, they gave estimates by median and quartile, which might have affected the heterogeneity. In addition, since some of the included studies did not describe all relevant patient characteristics, it was difficult to adjust for potentially confounding factors, such as comorbidity and treatment (including the use of anticoagulation or glucocorticoids). To maintain reliability of our conclusions, all low-quality studies were excluded. Finally, since retrospective studies were included in our meta-analysis, there was a risk of bias in the data collected.

In conclusion, our findings demonstrate that coagulopathy is associated with the severity of COVID-19 illness. D-dimer, PT, and FIB values are important parameters for evaluating the coagulopathy in COVID-19 patients. Since COVID-19 associated coagulopathy differs from disseminated intravascular coagulation, it is necessary to monitor closely the dynamics of coagulation parameters in COVID-19 patients.

## MATERIALS AND METHODS

### Search strategy

The protocol for this systematic review and meta-analysis has been registered in the International Platform of Registered Systematic Review and Meta-analysis Protocols (INPLSY) as INPLASY2020500049 (https://inplasy.com/). The articles included in the systematic review followed the Meta-analysis of Observational Studies in Epidemiology (MOOSE) guidelines (the checklist is shown in Supplementary Table 1), and were written in English and Chinese. The electronic databases Pubmed, Embase, Cochrane, WanFang Database, CNKI, and medRxiv were searched for reports published from December 1, 2019 to May 1, 2020, using a combination of the following keywords: "COVID-19" or "2019 novel coronavirus infection" or "SARS-CoV-2” and” characteristics” or” coagulopathy” or “coagulation”. Besides, we screened and conducted a manual search of the references listed in each article to obtain comprehensive results. The search was done independently by two authors (Xiaolin Zhang and Xue Yang). A third investigator (Hongmei Jiao) assisted with the resolution of any contradicting search results.

### Inclusion and exclusion criteria

The inclusion criteria were as follows: (1) Original studies focused on clinical characteristics of patients with COVID-19 including observational studies, case-control studies, cohort studies, and randomized control studies; (2) patients were categorized into less and more severe groups; (3) the coagulation parameters between groups were described. Exclusion criteria were as follows: (1) Non-original studies including commentaries, editorials, case reports, letters, meta-analysis, guidelines, and family-based studies; (2) same patients were enrolled in different studies; (3) patients were below 18 years old.

### Data extraction and quality assessment

Two investigators (Xiaolin Zhang and Xue Yang) independently extracted the data and evaluated their quality. Contradicting conclusions from the two investigators were resolved by a third investigator (Hongmei Jiao) or through a common consensus. Data extracted from each study included the study characteristics, demographic information, and the outcomes. We used the Newcastle-Ottawa scale (NOS), which included patient selection, study comparability, and three components of outcomes assessment to evaluate the quality of the original study. Low-quality articles (NOS<5) were excluded from this meta-analysis.

### Data analysis

All statistical analyses were performed via Stata 14.0 (Stata, College Station, TX, USA). For continuous variables, we calculated the standard mean difference (SMD) and the 95%CI. Heterogeneity among the studies was assessed using the Chi-squared and I^2^ tests. For studies that reported only median and range, CI, or interquartile range, we estimated means and SDs as described by Wan [43]. A random-effects model was used when either P<0.05 or I^2^>50% defined significant heterogeneity across the articles. Otherwise, we used the fixed-effects model. A p-value of less than 0.05 was considered significant. Sensitivity analysis was conducted to evaluate the stability of the results and determine the effect of individual study on pooled results. Evidence of publication bias was examined using Egger’s regression test for funnel asymmetry in addition to the visual inspection of funnel plots.

## Supplementary Material

Supplementary Figures

Supplementary Table 1

Supplementary Table 2

Supplementary Table 3
