# Supplementary material for: Coagulopathy in patients with COVID-19: a systematic review and meta-analysis
Source: Aging (Albany NY). 2020 Nov 24;12(24):24535–51. doi: 10.18632/aging.104138 (PMC7803569; doi:10.18632/aging.104138)
Supplement: Supplementary Figures [file aging-12-104138-s001.pdf]

SUPPLEMENTARY FIGURE

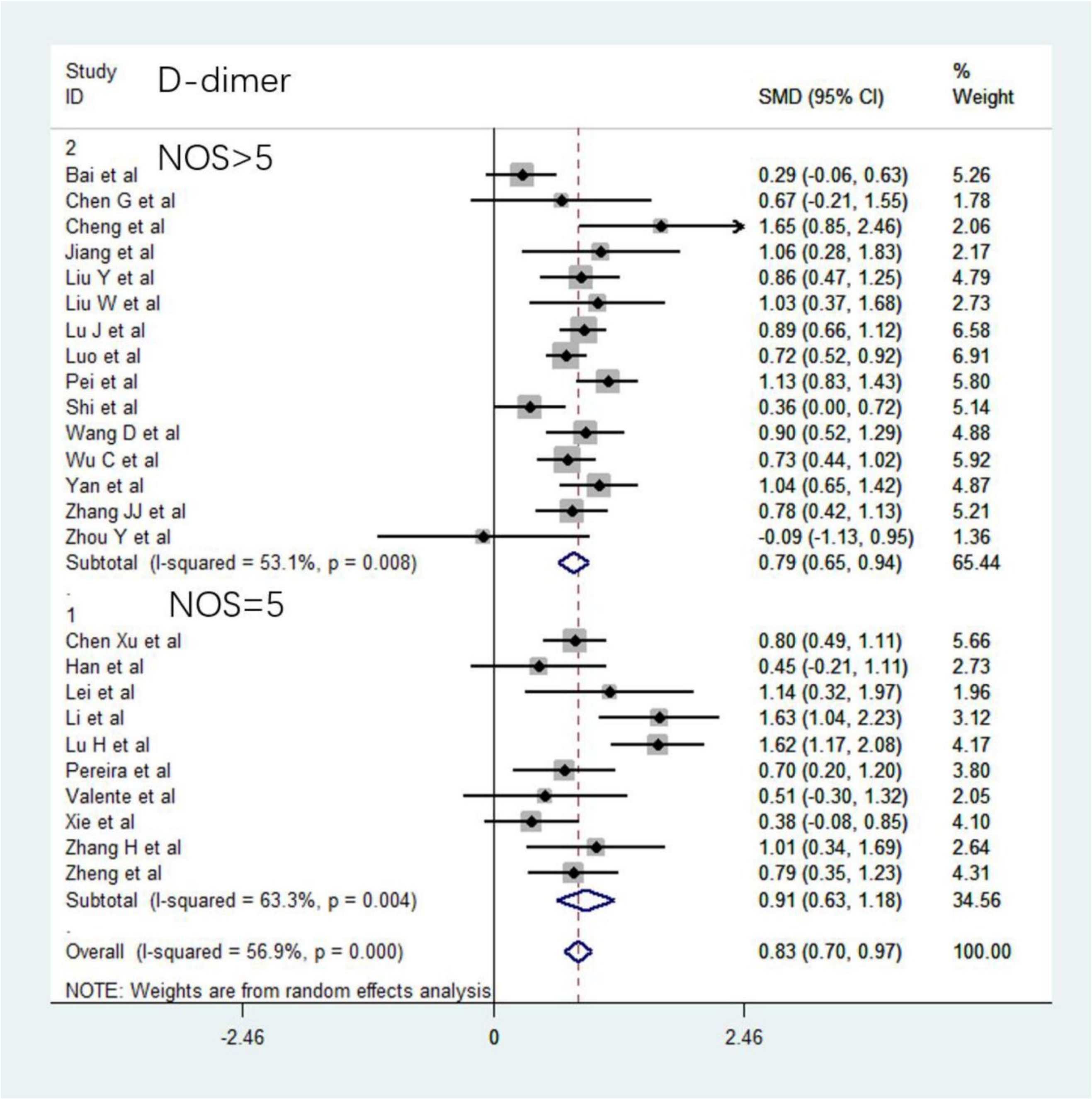

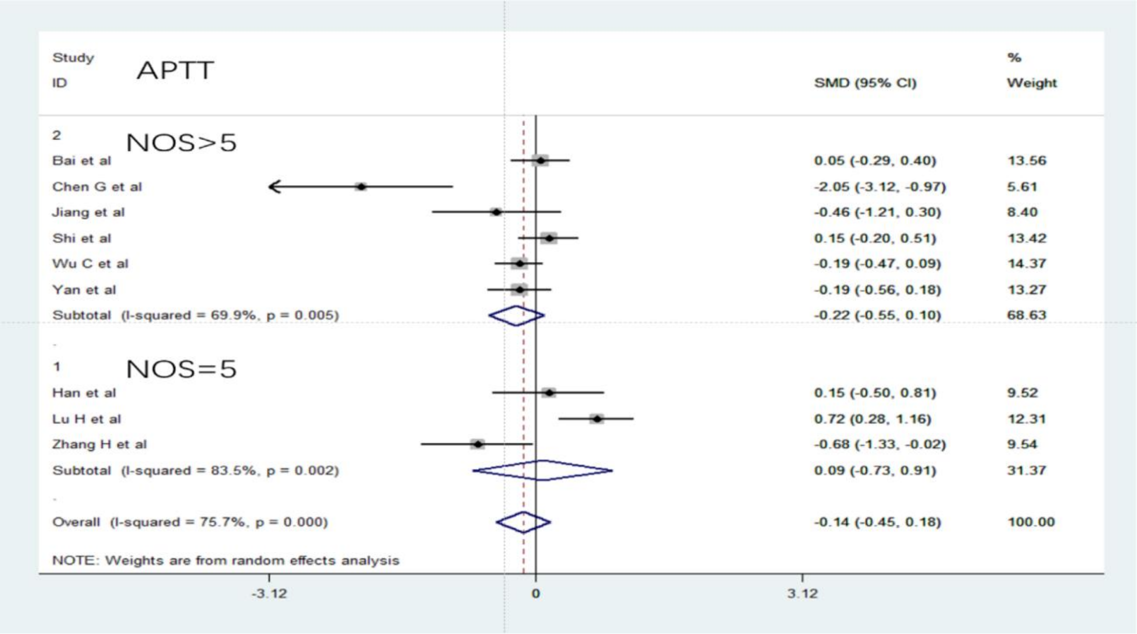
Study
ID
APTT
SMD (95% CI)
%
Weight
2
NOS>5
Bai et al 0.05 (-0.29, 0.40) 13.56
Chen G et al -2.05 (-3.12, -0.97) 5.61
Jiang et al -0.46 (-1.21, 0.30) 8.40
Shi et al 0.15 (-0.20, 0.51) 13.42
Wu C et al -0.19 (-0.47, 0.09) 14.37
Yan et al -0.19 (-0.56, 0.18) 13.27
Subtotal (I-squared = 69.9%, p = 0.005) -0.22 (-0.55, 0.10) 68.63
1
NOS=5
Han et al 0.15 (-0.50, 0.81) 9.52
Lu H et al 0.72 (0.28, 1.16) 12.31
Zhang H et al -0.68 (-1.33, -0.02) 9.54
Subtotal (I-squared = 83.5%, p = 0.002) 0.09 (-0.73, 0.91) 31.37
Overall (I-squared = 75.7%, p = 0.000) -0.14 (-0.45, 0.18) 100.00
NOTE: Weights are from random effects analysis
-3.12
0
3.12

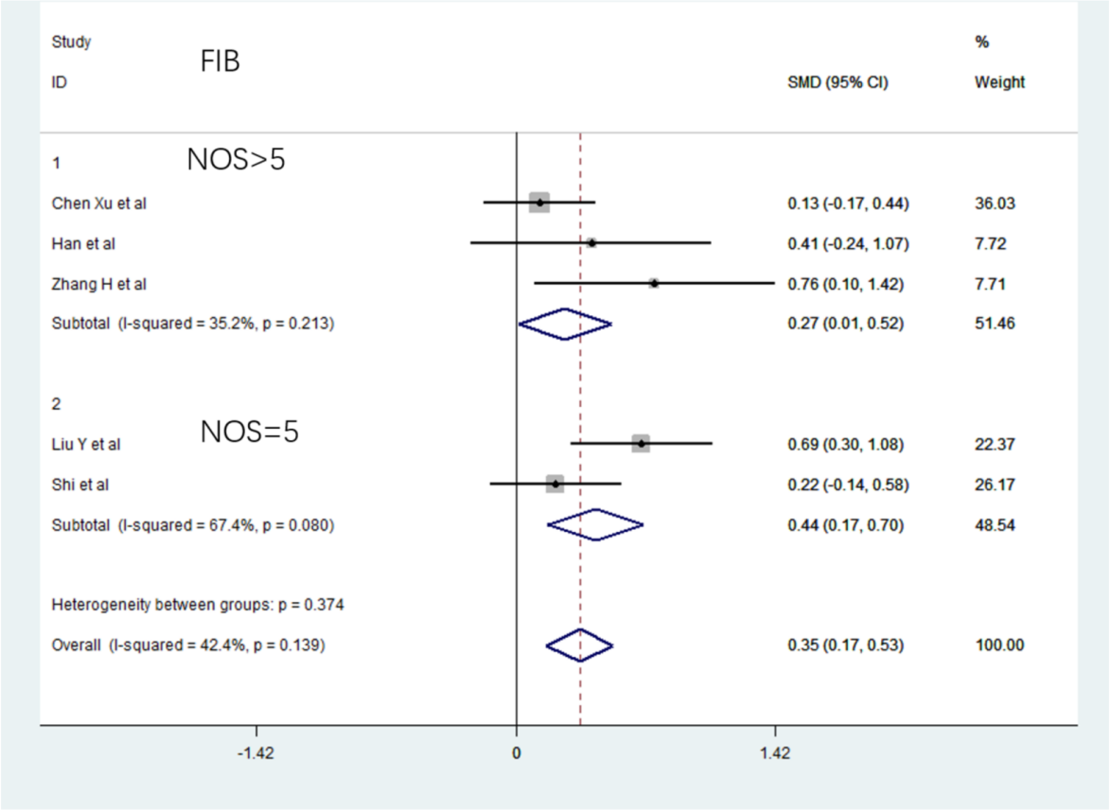
Study
ID
FIB
SMD (95% CI)
%
Weight
1
NOS>5
Chen Xu et al 0.13 (-0.17, 0.44) 36.03
Han et al 0.41 (-0.24, 1.07) 7.72
Zhang H et al 0.76 (0.10, 1.42) 7.71
Subtotal (I-squared = 35.2%, p = 0.213) 0.27 (0.01, 0.52) 51.46
2
NOS=5
Liu Y et al 0.69 (0.30, 1.08) 22.37
Shi et al 0.22 (-0.14, 0.58) 26.17
Subtotal (I-squared = 67.4%, p = 0.080) 0.44 (0.17, 0.70) 48.54
Heterogeneity between groups: p = 0.374
Overall (I-squared = 42.4%, p = 0.139) 0.35 (0.17, 0.53) 100.00
-1.42
0
1.42

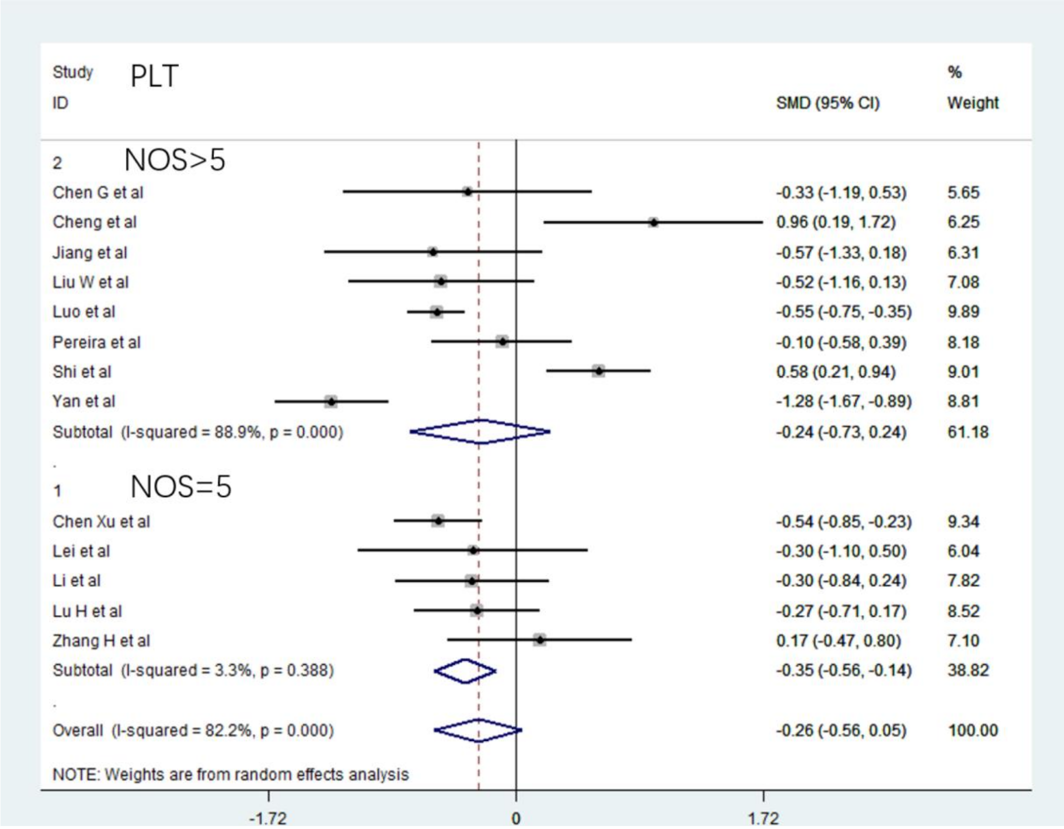

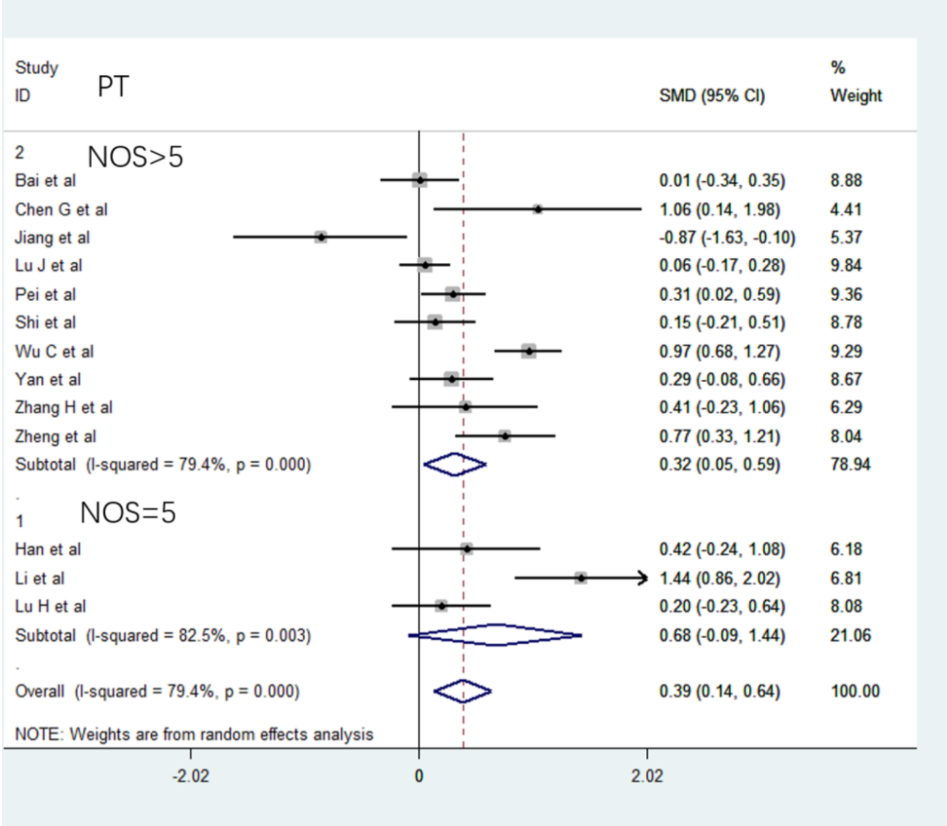

Supplementary Figure 1. Subgroup analyses of the association between D-dimer, PLT, PT, APTT in patients with COVID-19 stratified by disease severity according to NOS > 5 and NOS = 5.
