# Supplementary material for: Coagulopathy in patients with COVID-19: a systematic review and meta-analysis
Source: Aging (Albany NY). 2020 Nov 24;12(24):24535–51. doi: 10.18632/aging.104138 (PMC7803569; doi:10.18632/aging.104138)
Supplement: Supplementary Table 1 [file aging-12-104138-s002.doc]

**Supplementary Table 1. MOOSE Checklist.**

**MOOSE Checklist**

**Coagulopathy in patients with Coronavirus Disease 2019(COVID-19):a systematic review and meta-analysis**

Xiaolin Zhang*, Xue Yang*, Hongmei Jiao, Xinmin Liu

Department of Geriatrics, Peking University First Hospital

*These authors contributed equally to this work

Corresponding Author :

XinMin Liu

Peking University First Hospital,

Beijing 100034, China

Tel: +86-10-66110802

Fax: +86-10-66110802

E-mail: lxmgeriatric@126.com

| **Criteria** | | **Brief description of how the criteria were handled in the meta-analysis** |
| --- | --- | --- |
| **Reporting of background should include** | |  |
| √ | Problem definition | COVID-19 is associated with coagulopathy and a significant increased risk of thrombotic events. This meta-analysis aimed to explore coagulopathy on severity differences in patients with COVID-19. |
| √ | Hypothesis statement | Coagulation dysfunction was associated with severity of COVID-19. |
| √ | Description of study outcomes | The severity of patients with COVID-19. |
| √ | Type of exposure or intervention used | More severe patients with COVID-19. |
| √ | Type of study designs used | We included original studies including observational study, case-control studies, cohort studies and randomized control studies |
| √ | Study population | Adult patients with COVID-19. |
| **Reporting of search strategy should include** | |  |
| √ | Qualifications of searchers | The literature search was carried out independently by two investigators Xiaolin Zhang and Xue Yang. |
| √ | Search strategy, including time period included in the synthesis and keywords | We searched the electronic databased Pubmed, Embase, Cochrane, WanFang Database, CNKI, and medRxiv from December1,2019 to May 1,2020 using a combination of the following key words:"Covid-19" or "2019 novel coronavirus infection" or "SARS-CoV-2”and “characteristics” or “coagulopathy” or “coagulation”. |
| √ | Databases and registries searched | Pubmed, Embase, Cochrane, WanFang Database, CNKI, and medRxiv |
| √ | Search software used, name and version, including special features | We did not employ a search software. EndNote was used to merge retrieved citations and eliminate duplications. |
| √ | Use of hand searching | We hand-searched bibliographies of retrieved papers for additional references. |
| √ | List of citations located and those excluded, including justifications | Details of the literature search process are outlined in the flow chart. The citation list is available upon request. |
| √ | Method of addressing articles published in languages other than English | The language of literature included English and Chinese. We translated the Chinese literatures into English version. |
| √ | Method of handling abstracts and unpublished studies | We had contacted a few authors for unpublished studies on the association. |
| √ | Description of any contact with authors | We contacted authors who had conducted analysis of clinical characteristics of COVID-19, but they had not reported coagulation dysfunction. |
| **Reporting of methods should include** | |  |
| √ | Description of relevance or appropriateness of studies assembled for assessing the hypothesis to be tested | Detailed inclusion and exclusion criteria were described in the methods section. |
| √ | Rationale for the selection and coding of data | Data items extracted from each study included study characteristics, demographic information, and outcomes of interest. |
| √ | Assessment of confounding | There were strict inclusion criteria. Studies of low quality were excluded. |
| √ | Assessment of study quality, including blinding of quality assessors; stratification or regression on possible predictors of study results | We used the Newcastle-Ottawa scale (NOS),which includes patients selection, study comparability and outcomes assessment three components to evaluate the quality of the original study. Articles of low quality (NOS<5) were excluded from the meta-analysis. |
| √ | Assessment of heterogeneity | Heterogenity among the studies was assessed by the Chi squared and I2 tests. |
| √ | Description of statistical methods in sufficient detail to be replicated | Description of methods of meta-analyses, subgroup analysis and assessment of publication bias are detailed in the methods. |
| √ | Provision of appropriate tables and graphics | We included flow chart detailing the terms used for database search, summary table of included studies, summary table of meta-analysis and subgroup analysis, forest plot of all studies, funnel and Egger’s test diagram of all studies. |
| **Reporting of results should include** | |  |
| √ | Graph summarizing individual study estimates and overall estimate | Figure 2 |
| √ | Table giving descriptive information for each study included | Table 1 |
| √ | Results of sensitivity testing | Table 2 |
| √ | Indication of statistical uncertainty of findings | 95% confidence intervals were presented with all summary estimates, I2 values and results of sensitivity analyses |
| Reporting of discussion should include | |  |
| √ | Quantitative assessment of bias | Heterogeneity in strengths of the association due to most common biases in observational studies. |
| √ | Justification for exclusion | We excluded the studies with low quality. |
| √ | Assessment of quality of included studies | We discussed the results of the sensitivity analyses, and potential reasons for the observed heterogeneity. |
| Reporting of conclusions should include | |  |
| √ | Consideration of alternative explanations for observed results | We discussed that potential unmeasured confounders such as other chronic diseases may have caused residual confounding, but the measured factors that are correlated with such confounders would have mitigated the bias. |
| √ | Generalization of the conclusions | Coagulation dysfunction was associated with severity of COVID-19.D-dimer,PT,FIB were the dominate dominate parameters to evaluate coagulopathy in COVID-19. |
| √ | Guidelines for future research | We recommend future studies on the [dynamic](javascript:;) [change](javascript:;) of coagulation function in COVID-19. |
| √ | Disclosure of funding source | No separate funding was necessary for the undertaking of this systematic review. |
