# Supplementary material for: Coagulopathy in patients with COVID-19: a systematic review and meta-analysis
Source: Aging (Albany NY). 2020 Nov 24;12(24):24535–51. doi: 10.18632/aging.104138 (PMC7803569; doi:10.18632/aging.104138)
Supplement: Supplementary Table 2 [file aging-12-104138-s003.pdf]

Supplementary Table 2. Results of quality assessment using Newcastle-Ottawa Scale for observational studies.

	Selection				Comparability	Exposure			
**Study**	**Is the case definition adequate**	**Representativeness of the cases**	**Selection of controls**	**Definition of controls**	**Comparability of cases and controls on the basis of the design or analysis**	**Ascertainment of exposure**	**Same method of ascertainment for cases and controls**	**Non-Response rate**	**scores**
Bai et al	★	★	★	★			★	★	6
Chen G et al	★	★	★	★		★	★		6
Chen Xu et al	★	★	★	★			★		5
Cheng et al	★	★	★	★	★		★		6
Han et al	★	★	★	★	★	★	★		7
Jiang et al	★	★	★	★		★	★	★	7
Lei et al	★	★	★	★	★		★		6
Li et al	★	★	★	★	★	★	★		7
Liu Y et al	★	★	★	★	★	★	★		7
Liu W et al	★	★	★	★		★	★		6
Lu H et al	★	★	★	★			★		5
Lu J et al	★	★	★	★	★		★	★	7
Luo et al	★	★	★	★	★		★		6
Pei et al	★	★	★	★	★		★		6
Pereira et al	★	★	★	★			★	★	6
Shi et al	★	★	★	★			★	★	6
Valente et al	★	★	★	★			★		5
Wang D et al	★	★	★	★	★		★		6
Wu C et al	★	★	★	★	★		★	★	6
Xie et al	★	★	★	★	★		★		6
Yan et al	★	★	★	★			★	★	6
Zhang H et al	★	★	★	★			★		5
Zhang JJ et al	★	★	★	★		★	★	★	7
Zheng et al	★	★	★	★			★		5
Zhou Y et al	★	★	★	★			★		5
Lei S et al	★	★	★	★					4
Dan W et al	★		★	★					3
Cheng Q	★	★	★	★					4

Wan S	★	★	★	★			4
Huang C	★		★	★		★	4
Liu M	★	★	★	★			4
Cai Q et al	★	★		★	★		4
Zuo F et al	★	★	★	★			4
Zhan T et al	★	★	★	★			4
