# Supplementary material for: Coagulopathy in patients with COVID-19: a systematic review and meta-analysis
Source: Aging (Albany NY). 2020 Nov 24;12(24):24535–51. doi: 10.18632/aging.104138 (PMC7803569; doi:10.18632/aging.104138)
Supplement: Supplementary Table 3 [file aging-12-104138-s004.docx]

**Supplementary Table 3.** Main characteristics of the included studies in meta-analysis.

| **Author** | **Period** | **Country** | **Location** | **Language** | **Study design** | **Sample** | **Age [(mean ± SD), or median]** | **Male, number (%)** | **Severe definition** | **Outcome group, n (%)** | **Quality score** | **Reference** |
| --- | --- | --- | --- | --- | --- | --- | --- | --- | --- | --- | --- | --- |
| **Bai et al** | 2020.1.3-2020.2.13 | China | Wuhan | English | Retrospective  study | 133 | 52.82±12.59 | 66 (49.6%) | guidelines | Less severe 79 (59.4%)  More severe 54 (40.6%) | 6 | 9 |
| **Chen G et al** | 2019.12-2020.1.27 | China | Wuhan | English | Retrospective study | 21 | 56.0 (50.0, 65.0) | 17 (81.0%) | guidelines | Less severe 10 (47.6%)  More severe 11 (52.4%) | 6 | 10 |
| **Chen Xu et al** | 2020.1.23-2020.2.14 | China | Hunan | English | Retrospective study | 291 | 46.0(34.0, 59.0) | 145 (49.8%) | guidelines | Mild 29（10%）  Moderate 212 (72.8%)  Severe/critical 50 (17.2%) | 5 | 11 |
| **Cheng et al** | 2020.1.23-2020.3.10 | China | Anhui | English | Retrospective Study | 59 | 39.0 (30.0, 54.0) | 32 (54.2%) | guidelines | Mild 5 (8.5%)  Moderate 46 (78%)  Severe 8 (13.5%) | 6 | 12 |
| **Han et al** | 2020.1.31-2020.2.10 | China | Wuhan | English | Retrospective Study | 94 | NA | 48 (51%) | guidelines | Ordinary 49 (52.1%)  Severe 35 (37.2%)  Critical 10 (10.7%) | 5 | 13 |
| **Jiang et al** | 2020.1.23-2020.2.16 | China | Wuxi | English | Retrospective study | 55 | 45.0 (27.0,60.0) | 27 (49.1%) | guidelines | Non-severe 47 (85.5%)  Severe 8 (14.5%) | 6 | 14 |
| **Lei et al** | 2020.1.20-2020.2.3 | China | Chongqing | English | Retrospective study | 51 | 45.0 (34.0, 51.0） | 32(62.7%) | guidelines | Non-severe 44 (86.3%)  Severe 7 (13.7%) | 5 | 15 |
| **Li et al** | 2020.1.20-2020.2.27 | China | Hunan | Chinese | Retrospective study | 80 | 47.5 (3-90) | 40（50%） | guidelines | Non-severe 63 (78.8%)  Severe 17 (21.2%) | 5 | 16 |
| **Liu Y et al** | 2020.1.2-2020.2.1 | China | Wuhan | English | Retrospective study | 109 | 55 (43-66) | 59 (54.1) | ARDS | Non-ARDS (n = 56)  ARDS (n = 53) | 6 | 17 |
| **Liu W et al** | 2020.12.30-2020.1.15 | China | Wuhan | English | Retrospective study | 78 | 38 (33, 57) | 39 (50.0) | guidelines | Improvement/stabilization  67 (85.9%)  Progression 11(14.1%) | 6 | 18 |
| **Lu H et al** | 2020.1.20-2020.2.9 | China | Shanghai | English | Retrospective, cohort study | 265 | NA | NA | guidelines | \| Mild-moderate 243 (91.7%)  Severe-critically 22 (8.3%) \|  \| \| --- \| --- \| | 5 | 19 |
| **Lu J et al** | 2020.1.21-2020.2.5 | China | Wuhan | English | Retrospective cohort study | 577 | 55 (39, 66) | 254 (44.0) | guidelines | Mild 338 (77.2%)  Severe 100 (22.8%) | 6 | 20 |
| **Luo et al** | 2020.1.30-2020.2.25 | China | Wuhan | English | Retrospective  cohort study | 403 | 56 (39, 68) | 193 (47.9%) | guidelines | Ordinary 198 (49.1%)  Severe or critical 205 (50.9%) | 6 | 21 |
| **Pei et al** | 2020.1.28-2020.2.9 | China | Wuhan | English | Retrospective  cohort study | 333 | 56.36±13.4 | 182(54.7%) | guidelines | Moderate 144 (43.2%)  Severe 133 (39.9%)  Critically Ill 56 (16.8%) | 6 | 22 |
| **Pereira et al** | 2020.3.13-2020.4.3 | American | New York | English | Retrospective cohort study | 90 | 57 (46, 68) | 53 (59%) | ICU | Mild/moderate disease 63 (70%)  Severe disease 27 (30%) | 5 | 23 |
| **Shi et al** | 2020.1.23-2020.3.7 | China | Shanxi | English | Retrospective cohort study | 134 | 46(34, 58) | 65(48.5%) | guidelines | Non-severe 88 (65.7%)  Severe 46 (34.2%) | 6 | 24 |
| **Valente et al** | 2020.3.13-2020.4.13 | Mexico | Mexico | English | Descriptive case series report | 33 | 60.6±12.68 | 23 (69.7%) | guidelines | Severe Pneumonia 25 (75.8%)  Critical Pneumonia 8 (24.2%) | 5 | 25 |
| **Wang D et al** | 2020.1.18-2020.2.28 | China | Wuhan | English | Descriptive case series report | 115 | 59 (40-67) | 58 (50.4%) | guidelines | Noncritical 60 (52.2%)  Critical 55 (47.8%) | 6 | 26 |
| **Wu C et al** | 2019.12.25-2020.1.26 | China | Wuhan | English | Retrospective cohort study | 201 | 51 (43-60) | 128 (63.7%) | ARDS | Without ARDS 117 (58.2%)  With ARDS 84 (41.8%) | 6 | 27 |
| **Xie et al** | 2020.2.2-2020.2.23 | China | Wuhan | English | Retrospective cohort study | 79 | 60.0 (48.0-66.0) | 44 (55.7%) | guidelines | Moderate 51 (64.6%)  Severe 28 (35.4%) | 5 | 28 |
| **Yan et al** | 2020.1.22-2020.3.14 | China | Hainan | English | Retrospective cohort study | 168 | 51 (36-62) | 81 (48.2%) | guidelines | Non-severe132 (78.6%)  Severe 36 (21.4%) | 6 | 29 |
| **Zhang H et al** | 2020.1.11-2020.1.28 | China | Chongqing | English | Retrospective cohort study | 43 | NA | 22 (51.2%) | guidelines | Mild 29 (67.4%)  Severe 14 (32.6%) | 5 | 30 |
| **Zhang JJ et al** | 2020.1.16-2020.2.3 | China | Wuhan | English | Retrospective cohort study | 140 | 57 (25-87) | 71 (50.7%) | guidelines | Non-severe 82 (58.6%)  Severe 58 (41.4%) | 7 | 31 |
| **Zheng et al** | 2020.1.16-2020.2.20 | China | Chengdu | English | Retrospective cohort study | 99 | 49.40±18.45 | 51 (51.5%) | guidelines | Noncritical 67 (67.7%)  Critical 32 (32.3%) | 5 | 32 |
| **Zhou Y et al** | 2020.1.28-2020.3.2 | China | Nanjing | English | Retrospective cohort study | 21 | NA | 6(35.3%) | guidelines | \| Non-aggravation 12 (70.6%)  Aggravation 5 (29.4%) \| Aggravation group (n=5) \| \| --- \| --- \| | 6 | 33 |
